# PlaNet-S: an Automatic Semantic Segmentation Model for Placenta Using U-Net and SegNeXt

**DOI:** 10.1007/s10278-025-01549-9

**Published:** 2025-05-27

**Authors:** Isso Saito, Shinnosuke Yamamoto, Eichi Takaya, Ayaka Harigai, Tomomi Sato, Tomoya Kobayashi, Kei Takase, Takuya Ueda

**Affiliations:** 1 Department of Clinical Imaging, Tohoku University Graduate School of Medicine, Sendai, Miyagi Japan; 2 AI Lab, Tohoku University Hospital, 1-1 Seiryo-Machi, Aoba-Ku, Sendai, Miyagi 980-8574 Japan; 3 Department of Diagnostic Radiology, Tohoku University Hospital, 1-1 Seiryo-Machi, Aoba-Ku, Sendai, Miyagi 980-8574 Japan; 4 Department of Diagnostic Radiology, Tohoku University Graduate School of Medicine, 2-1 Seiryo-Machi, Aoba-Ku, Sendai, Miyagi 980-8575 Japan; 5 Department of Radiology, Tohoku Medical and Pharmaceutical University, 1-15-1 Fukumuro, Miyagino, Sendai, Miyagi 983-8536 Japan

**Keywords:** Deep learning, Placenta, Magnetic resonance imaging, Automatic semantic segmentation, Vision transformer

## Abstract

This study aimed to develop a fully automated semantic placenta segmentation model that integrates the U-Net and SegNeXt architectures through ensemble learning. A total of 218 pregnant women with suspected placental abnormalities who underwent magnetic resonance imaging (MRI) were enrolled, yielding 1090 annotated images for developing a deep learning model for placental segmentation. The images were standardized and divided into training and test sets. The performance of Placental Segmentation Network (PlaNet-S), which integrates U-Net and SegNeXt within an ensemble framework, was assessed using Intersection over Union (IoU) and counting connected components (CCC) against the U-Net, U-Net + + , and DS-transUNet. PlaNet-S had significantly higher IoU (0.78, SD = 0.10) than that of U-Net (0.73, SD = 0.13) (*p* < 0.005) and DS-transUNet (0.64, SD = 0.16) (*p* < 0.005), while the difference with U-Net + + (0.77, SD = 0.12) was not statistically significant. The CCC for PlaNet-S was significantly higher than that for U-Net (*p* < 0.005), U-Net + + (*p* < 0.005), and DS-transUNet (*p* < 0.005), matching the ground truth in 86.0%, 56.7%, 67.9%, and 20.9% of the cases, respectively. PlaNet-S achieved higher IoU than U-Net and DS-transUNet, and comparable IoU to U-Net + + . Moreover, PlaNet-S significantly outperformed all three models in CCC, indicating better agreement with the ground truth. This model addresses the challenges of time-consuming physician-assisted manual segmentation and offers the potential for diverse applications in placental imaging analyses.

**Supplementary Information:**

The online version contains supplementary material available at 10.1007/s10278-025-01549-9.

## Introduction

As a critical link between the mother and fetus, the placenta is essential for both pregnancy success and fetal growth [1]. Abnormalities in placental function may pose severe risks to both maternal and fetal health, potentially resulting in irreversible harm or life-threatening situations. Conditions such as placenta previa [2], placenta accreta spectrum (PAS) [3], and placental insufficiency [4, 5] are among the most common reasons for imaging evaluations during pregnancy. Assessment of the placenta is crucial for understanding placental structure, function, and development and to identify strategies to optimize pregnancy outcomes [4, 6, 7].

In placental imaging, the location, shape, and volume of the placenta are important factors for identifying abnormalities. Ultrasonography is the primary imaging modality for monitoring fetal health and development. Magnetic resonance imaging (MRI)–based evaluation of the placenta provides more detailed anatomical and functional information, especially for complex cases or posteriorly located placentas where ultrasound application is limited [7, 8]. Several studies have reported the usefulness of placental segmentation using MRI for monitoring conditions that can lead to pregnancy and birth complications, such as PAS, fetal growth restriction, and potential intrauterine fetal demise [9–11]. Despite its utility, manual segmentation in MRI is time-consuming and exhibits high inter- and intra-observer variability [12].

Deep learning (DL)–based approaches, especially those based on the convolutional neural network (CNN) architecture, have demonstrated a strong capability for the fast and automatic segmentation of medical images with high accuracy [13]. U-Net is a representative CNN architecture specifically designed for image-segmentation tasks [14]. Although several researchers have proposed automatic segmentation methods for the human placenta using the U-Net architecture [15, 16], U-Net-based segmentation models still require improvement in terms of robustness and generalizability. Recently, the Vision Transformer (ViT) was introduced as a novel architecture for DL-based image analysis [17]. ViT was adapted from the Transformer model originally designed for natural language processing [18]. Whereas CNN-based models predominantly rely on local textures in images [19, 20], ViT-based models emphasize the global shape of objects for image recognition [21]. Although ViT-based models have the potential to outperform CNN-based DL models in the field of semantic segmentation when trained on large datasets [17], their performance is heavily dependent on the amount of data [22]. As large-scale data are not always available in medical research, these transformers may not achieve optimal performance in such medical contexts.

SegNeXt (a simple convolutional network architecture for semantic segmentation), proposed by Guo et al., is a streamlined CNN architecture designed specifically for semantic segmentation [23]. To address the abovementioned limitations of ViT-based models, SegNeXt was designed to incorporate multiscale convolutional attention to encode contextual information, emphasizing cost-effective convolutional operations. By emulating the ViT mechanism and embedding it within a CNN framework, SegNeXt enhances its performance while concurrently optimizing computational expenses [23].

Recently, there were several studies assess adverse pregnancy outcome using MRI. For example, Gibbins et al. [24] revealed the possibility that MRI placental volume can be used as assessment of placental insufficiency. Zhu et al. [25] utilized radiomics model as automated diagnostic model of PAS based on MRI. Wu et al. [26] utilized both clinical and radiomic features, and built nomogram predicting the risk for pregnancies with postpartum hemorrhage (PPH). Given these considerations, the need for the automated semantic segmentation model for MRI images is increasing. This study aimed to develop a fully automated semantic placenta segmentation model by integrating ensemble learning techniques with the U-Net and SegNeXt architectures to enhance the reliability and precision of placental tissue delineation in MRI.

## Materials and Methods

### Patient Selection and Enrollment

In this study, we included placental MRI scans performed at our hospital between January 2004 and December 2021. Single-shot T2-weighted images from 218 patients with suspected placental abnormalities were analyzed. Ninety-five patients were diagnosed with placenta previa, 55 with placenta accreta spectrum, and 52 with both. This retrospective study was approved by the institutional review board (IRB no: 2023–1–629), and the requirement for informed consent from patients was waived.

### MRI Equipment and Parameters

MRI in this study was performed using the following four MRI scanners: 1.5 T-Achieva (Philips Medical Systems, Best, the Netherlands), 3 T-MAGNETOM Vida (Siemens Healthcare, Erlangen, Germany), 1.5 T-MAGNETOM VISION plus (Siemens Healthcare, Erlangen, Germany), and 3 T-Vantage Titan 3 T (Cannon Medical Systems, Tokyo, Japan). T2-weighted imaging of placenta was obtained using single-shot fast spin-echo T2-weighted sequences with repetition time (TR), ∞ ms; echo time (TE), 64–120 ms; acquisition matrix, 178 × 224–287 × 384; field of view (FOV), 263 × 300–420 × 420; and slice thickness, 4–10 mm.

### Image Selection and Annotation

A radiologist with 12-year expertise in gynecological imaging selected five consecutive images in which the placenta was clearly visible. Consequently, our dataset comprised 1090 images from 218 patients. For each image, a radiologist annotated the placental region using a custom-developed, iPad-based annotation application.

### Preprocessing and Image Dataset

To standardize the resolution of all MR images to 256 × 256 pixels, each image was resized based on its longest dimension, that is, height or width. The images were scaled such that the longest dimension was 256 pixels while preserving the original aspect ratio. For images that did not form a perfect square after resizing, the original image was centrally placed and the shorter dimension was symmetrically padded with black pixels, ensuring the desired 256 × 256 pixel size. Our original dataset, comprising 1090 images, was divided into training and test datasets in an 8:2 ratio, resulting in 875 and 215 images for the training and test datasets, respectively. The dataset was divided based on the patients to avoid image overlaps from the same patient between the training and test datasets, thus ensuring that all five MR images from a single patient were allocated to one of the training and test datasets.

### Model Architecture of Placental Segmentation Network (PlaNet-S)

**Fig. 1:**
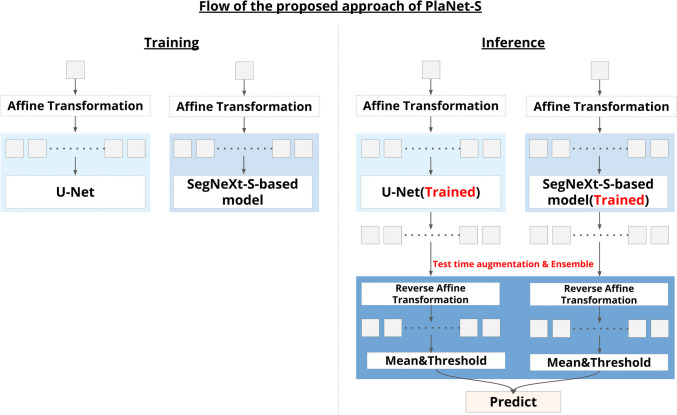
Flow of the proposed approach of PlaNet-S. The Placental Segmentation Network (PlaNet-S) is a specialized deep learning approach tailored for precise placental segmentation in MR imaging. The proposed model employs ensemble learning by integrating U-Net and SegNeXt. The process begins with an affine transformation applied to the input to create 200 variants. The 200 variants were distributed across two distinct models, each generating 100 outputs. After processing, the model predictions were reverted to their original spatial alignment using the inverse of the initial transformation. Predictions that met a specific threshold were combined to yield the final output from the union of the model results

In this study, a specialized DL model named the Placental Segmentation Network (PlaNet-S) is proposed for performing accurate segmentation of the placenta in MR imaging. Figure 1 illustrates the structural design and ensemble learning approach inherent in the PlaNet-S architecture. PlaNet-S employs ensemble-learning techniques that integrate the U-Net and SegNeXt architectures (Fig. 1) [14, 23]. Specifically, each model generates segmentation probability maps, which are then averaged to produce a single prediction map per model. A threshold of 0.5 is applied to these averaged maps to obtain binary segmentation results. Finally, a logical OR operation is performed between the two binary maps—if either model assigns a voxel to the placenta (label value of 1), that voxel is included in the final segmentation.

**Fig. 2:**
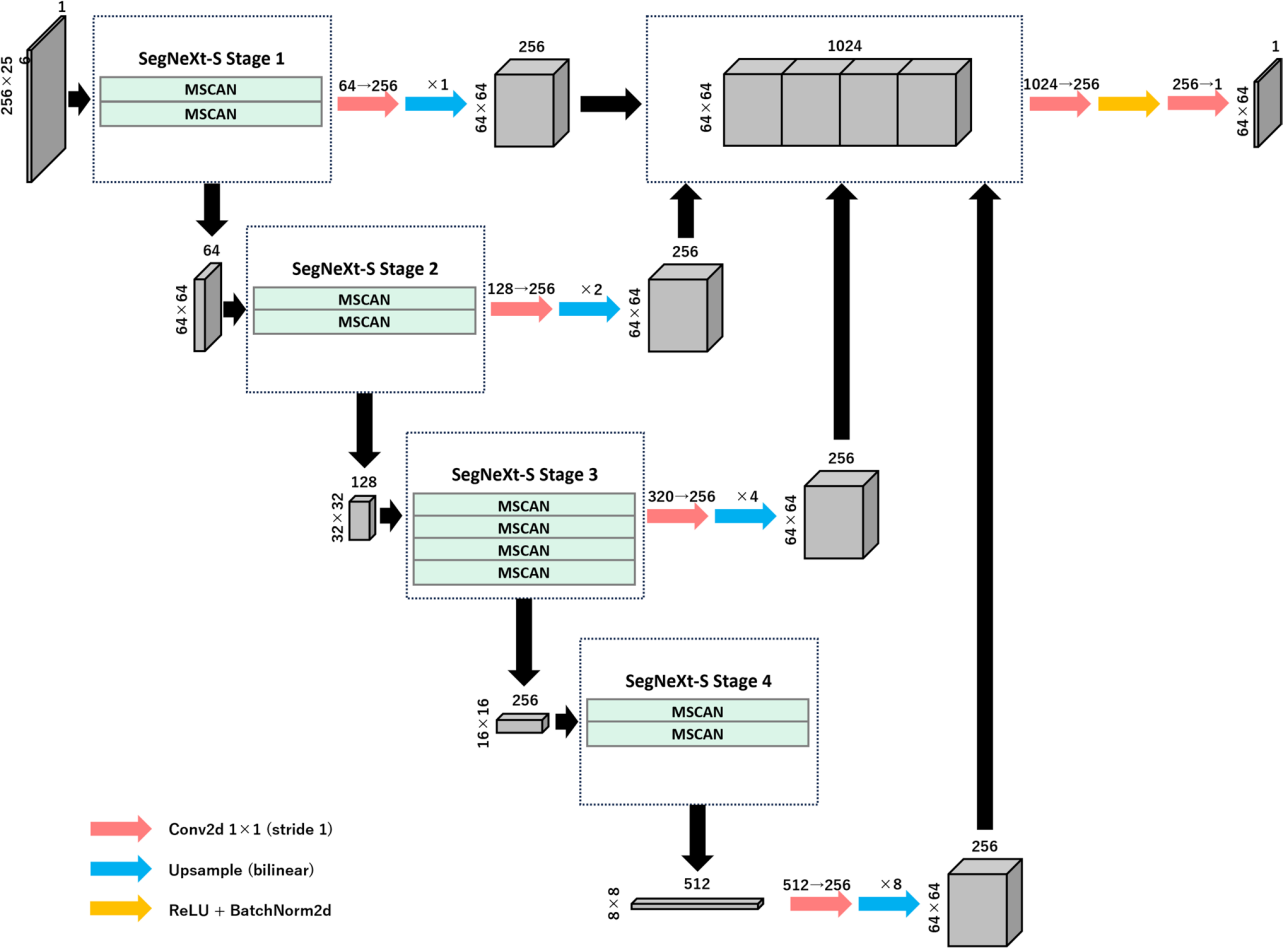
SegNeXt-S-based model. The structure progresses through stages 1–4, with each stage depicting a unique facet of the SegNeXt-S framework. The numerical annotations indicated varying complexities, possibly corresponding to the number of feature channels or neurons. The feature maps generated using the SegNeXt Encoder were transitioned to 256 channels and subsequently fused to form a 64 × 64 × 1024 feature volume. After two convolutional stages, the data were up-sampled, culminating in a final resolution of 256 × 256 × 1 pixels. The SegNeXt-S encoder is adeptly matched with a multilayer perceptron decoder, mirroring the design patterns in models such as SegFormer

Figure 2 presents part of the architectural design of the PlaNet-S model, which is deeply rooted in the SegNeXt variant. The PlaNet-S model employs the multi-scale convolutional attention (MSCAN) encoder, as adopted in SegNeXt, which is structured into four hierarchical stages with decreasing spatial resolutions (H/4 × W/4, H/8 × W/8, H/16 × W/16, and H/32 × W/32). Initially, the input image is processed by a stem convolution that down-samples it to H/4 × W/4. In each of the subsequent three stages, a down-sampling block (a 3 × 3 convolution with stride 2 and batch normalization) is applied, followed by a series of MSCAN building blocks that utilize batch normalization to enhance segmentation performance.

The loss function (L) for PlaNet-S is defined in Eq. (1). LBCE represents binary cross-entropy. LIoU is defined by Eq. (2) using Intersection over Union (IoU): The IoU is the ratio of the overlap area between the predicted and true bounding boxes and their union area, which can be used to quantify the accuracy of the segmentation [27]. Higher IoU scores indicated more accurate predictions of the true bounding boxes.

1 $$L={L}_{BCE}+{L}_{IoU}$$

2 $${L}_{IoU}=1-IoU$$

### Training Step

During the PlaNet-S training step, an affine transformation was utilized for data augmentation. Three elements of affine transformation were used: rotation, ranging from − 45 to + 45 degrees; shift, ranging from 0 to 1 in each of the (*x*, *y*) axes; and scaling, ranging from 0.8 to 1, with all parameters being randomly applied.

Adaptive moment estimation (Adam) was used as the optimizer. The learning rate, β1, β2, and ε were set to 1e − 3, 0.9, 0.999, and 1e − 8, respectively. A mini-batch size and number of epochs of 8 and 100 were used, respectively.

PlaNet-S was implemented using an NVIDIA Quadro RTX 8000 machine. The operating system was Ubuntu 18.04.5 long-term support (LTS): Xenial Xerus. All analyses were performed using Python, version 3.7.3 (Python Software Foundation, http://www.python.org). Pytorch version 1.9.5 was used as the deep-learning framework.

### Test-Time Augmentation

Test-time augmentation (TTA) was also applied for data augmentation during both the validation and testing phases. Although data augmentation is typically employed during the training step, TTA adopts a similar approach to improve the robustness toward the test and validation steps [28]. Mathematically, given an input image x, transformations 1, 2, … are applied to produce a set of transformed images × 1, × 2, …. For each transformed image xi, model M provides the prediction pi. The final prediction, P, is then computed by averaging the individual predictions.

In terms of formula:

3 $${p}_{i}=M({\phi }_{i}\left(x\right))$$

4 $$P= \frac{1}{N}\sum\nolimits_{i=1}^{N}{p}_{i}$$

### Validation Step

To mitigate the potential for overfitting the DL model, fivefold cross-validation was conducted. From each fold, the model that achieved the IoU score was selected as the definitive model. To enhance the robustness during validation, TTA was applied to the validation step.

### Test Step

For the segmentation task, the final prediction of PlaNet-S was assessed using a test set comprising 43 cases, each containing five images, yielding 215 images. The IoU was then calculated for each set of predictions. The TTA was applied during the test step to enhance the robustness of the model.

### Comparison of Model Performance of PlaNet-S, Traditional U-Net, U-Net + +, and DS-transUNet

To assess the performance of PlaNet-S, its predictions were compared with those of the traditional segmentation model, U-Net and U-Net + +, as well as the transformer-based benchmark model, DS-transUNet, using the same dataset. The IoU and Counts of Connected Components (CCC) were employed as criteria for performance assessment. The IoU, a well-established metric for assessing segmentation accuracy, quantifies the overlap between the predicted and true bounding boxes relative to their combined area. A higher IoU value typically indicates a more accurate depiction of placental segmentation. Additionally, we adopted as an auxiliary measure of segmentation precision. The CCC delineates distinct segmented regions in an image. Each connected component epitomizes a cluster of pixels or voxels either directly or indirectly, denoting discrete entities within the segmented image. Segmentation was considered more accurate when no difference was observed between the CCC calculated from the ground-truth annotation and that derived from the segmentation of the deep learning model. A paired *t*-test was used to assess the statistical significance of the difference in the IoU among PlaNet-S, U-Net, U-Net + +, and DS-transUNet. The Wilcoxon signed-rank test was used to assess the statistical significance of the differences in the CCC among PlaNet-S, U-Net, U-Net + +, and DS-tranUNet, excluding pairs with identical results. Although the nominal significance level was set at 0.01, it was adjusted to 0.003 for both tests based on the Bonferroni correction for multiple comparisons.

### Ablation Study for Component Effectiveness in PlaNet-S

In the ablation study, the abbreviations A and T denote the use of data augmentation and test time augmentation (TTA), respectively. To evaluate the impact of these augmentations, four configurations of PlaNet-S were compared: PlaNet-S (A–T–), PlaNet-S (A + T–), PlaNet-S (A-T +), and PlaNet-S (A + T +), with the latter representing the proposed method. The performance differences were assessed using the IoU and CCC metrics. Paired *t*-tests were performed to determine the statistical significance of the IoU differences, and Wilcoxon signed-rank tests were used for the CCC comparisons, with a Bonferroni-adjusted significance threshold of 0.003 (from a nominal *α* of 0.01).

## Results

### Model Performance of PlaNet-S, Traditional U-Net, U-Net + +, and DS-transUNet

**Fig. 3:**
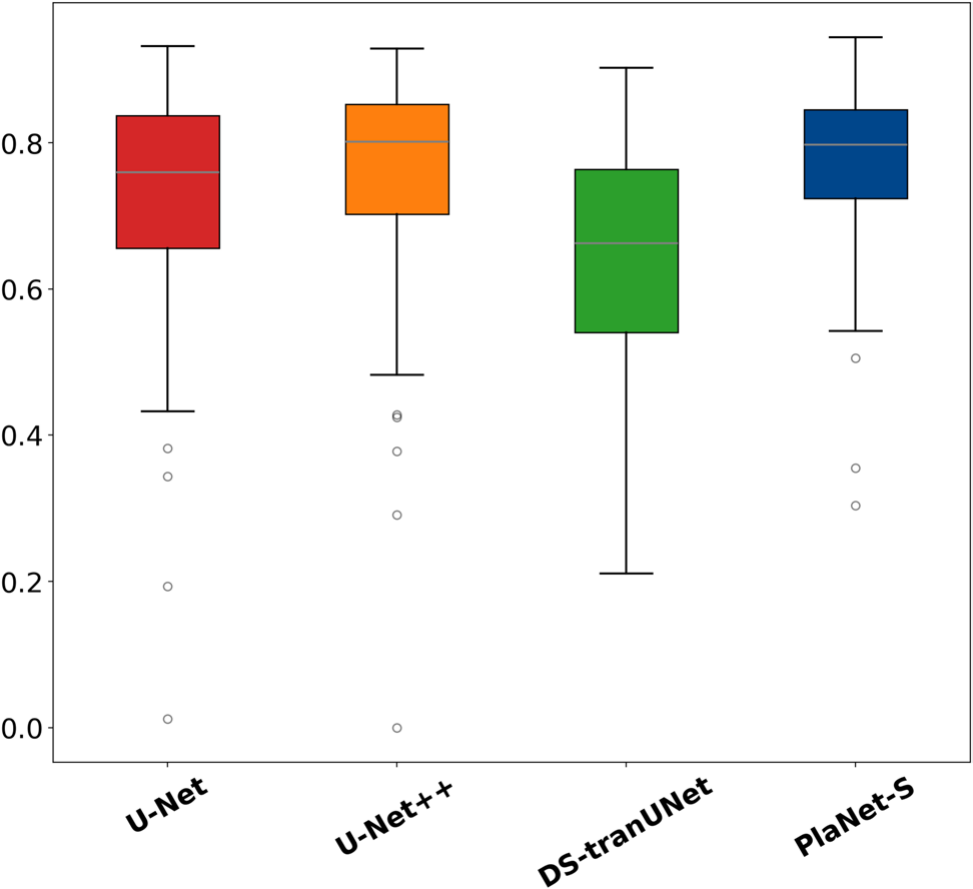
IoU (Intersection over Union) box plot for U-Net, U-Net + +, DS-transUNet, and PlaNet-S. The distribution of prediction accuracy, as represented by the IoU, for U-Net, U-Net + +, DS-transUNet, and PlaNet-S, is depicted in this box plot. U-Net, U-Net + +, DS-transUNet, and PlaNet-S showed mean IoU values of 0.73 (SD = 0.13), 0.77, (SD = 0.12), 0.64 (SD = 0.16), and 0.78 (SD = 0.10), respectively. The IoU for PlaNet-S was significantly higher than that for U-Net (*p* < 0.003) and DS-transUNet (*p* < 0.003), as determined by paired *t*-tests with the Bonferroni-adjusted significance level; however, the difference between PlaNet-S(A + T +) and U-Net + + was not statistically significant (*p* = 0.07)

Figure 3 shows box plots depicting the IoU for U-Net, U-Net + +, DS-transUNet, and PlaNet-S which showed means of 0.73 (SD = 0.13), 0.77 (SD = 0.12), 0.64 (SD = 0.16), and 0.78 (SD = 0.10), respectively (Table 1). The IoU for PlaNet-S was significantly higher than that for U-Net (*p* < 0.003) and DS-transUNet (*p* < 0.003), as determined by the paired t-tests with the Bonferroni-adjusted significance level; however, the difference between PlaNet-S(A + T +) and U-Net + + was not statistically significant (*p* = 0.07).

**Table 1:** Intersection over Union and the absolute differences in the Counts of Connected Components among U-Net, U-Net + +, DS-transUNet, and PlaNet-S

	IoU (mean ± SD)	CCC: matching the ground truth (%)
U-Net	0.73 ± 0.13	56.7
U-Net + +	0.77 ± 0.11	67.9
DS-transUNet	0.64 ± 0.16	20.9
PlaNet-S	0.78 ± 0.10	86.0

*IoU*; Intersection over Union, *CCC*; Counts of Connected Components

**Fig. 4:**
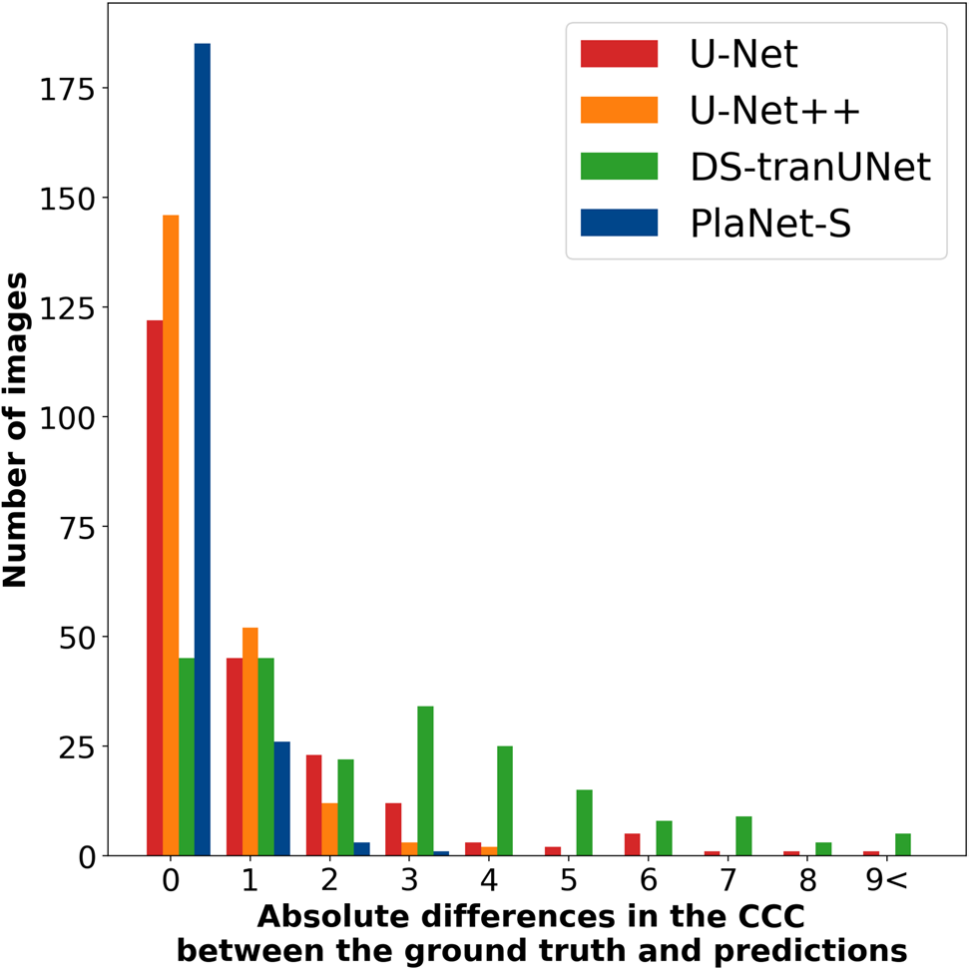
Bar plot comparing the absolute differences in the Counts of Connected Components (CCC) between ground truth and predictions for U-Net, U-Net + +, DS-transUNet, and PlaNet-S. The bar plot depicts the absolute differences between the ground-truth number of connected components and those inferred from the segmentation results of the three models. The *x*-axis represents the absolute differences in the CCC between the ground truth annotations and segmentations performed by the DL models, whereas the *y*-axis indicates the cumulative number of patients corresponding to those differences. The CCC for PlaNet-S was significantly lower than those for the other models (all *p* < 0.003), as determined by Wilcoxon signed-rank tests with the Bonferroni-adjusted significance level

Figure 4 shows a bar plot depicting the absolute difference between the ground-truth number of Connected Components and the inferred Connected Components calculated from the results of U-Net, U-Net + +, DS-transUNet, and PlaNet-S. U-Net, U-Net + +, DS-transUNet, and PlaNet-S achieved segmentation with an absolute difference of zero in the number of connected components for 122, 146, 45, and 185 test images, representing, 56.7%, 67.9%, 20.9%, and 86.0% of the dataset, respectively (Table 1). The CCC for PlaNet-S was significantly smaller than that for the other models (all *p* < 0.003), as determined by the Wilcoxon signed-rank tests with the Bonferroni-adjusted significance level.

### The Ablation Study Revealing the Component Effectiveness

**Fig. 5:**
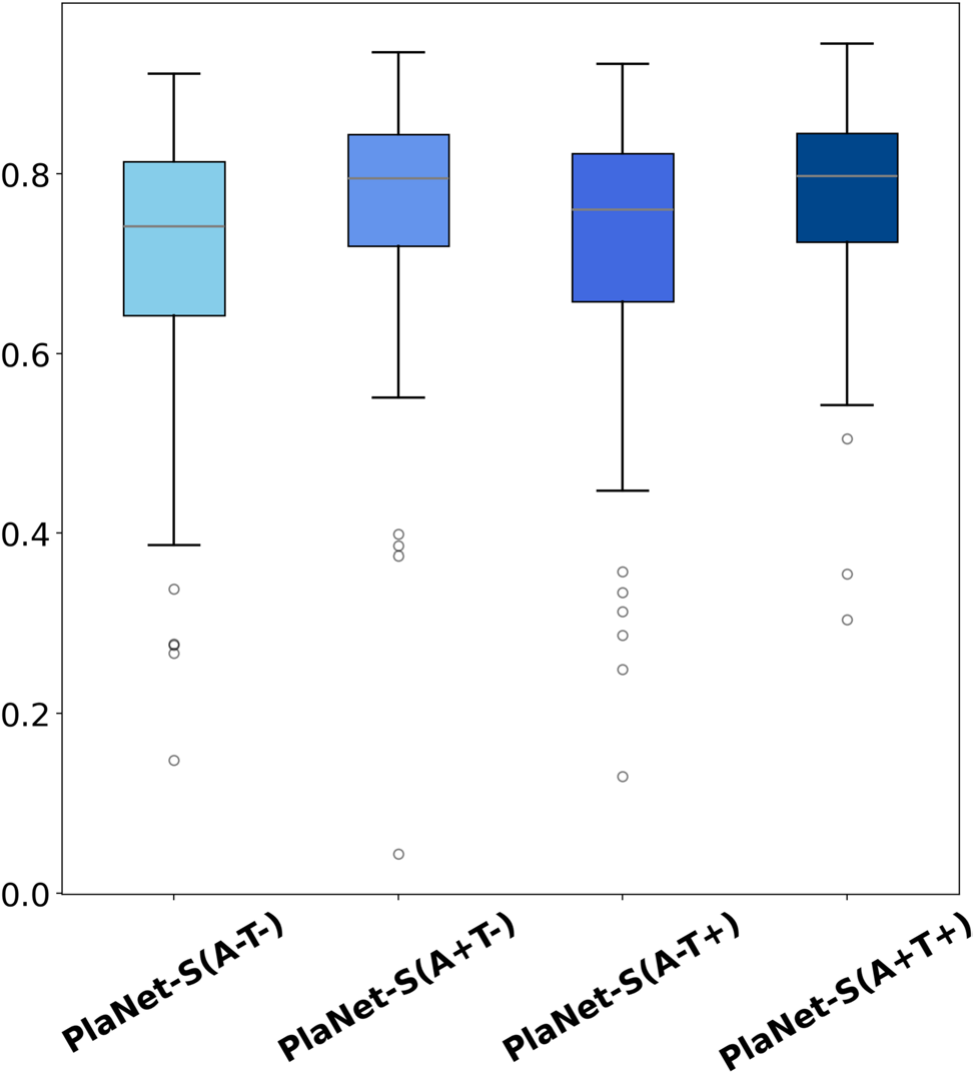
IoU (Intersection over Union) box plot for PlaNet-S (A–T–), PlaNet-S (A + T–), PlaNet-S (A–T +), and PlaNet-S (A + T +). The distribution of prediction accuracy, as represented by the IoU, for PlaNet-S (A–T–), PlaNet-S (A + T–), PlaNet-S (A-T +), and PlaNet-S (A + T +), which is the proposed method, is depicted in this box plot. PlaNet-S (A–T–), PlaNet-S (A + T–), PlaNet-S (A-T +), and PlaNet-S (A + T +) showed mean IoU values of 0.71 (SD = 0.14), 0.77 (SD = 0.11), 0.73 (SD = 0.14), and 0.78 (SD = 0.10), respectively. The IoU for PlaNet-S (A + T +) was significantly higher than that for PlaNet-S (A–T–) (*p* < 0.003) and PlaNet-S (A–T +) (*p* < 0.003), as determined by paired t-tests with the Bonferroni-adjusted significance level; however, the difference between PlaNet-S (A + T +) and PlaNet-S (A + T–) was not statistically significant (*p* = 0.02)

Figure 5 shows a comparison of the IoU in an ablation study among four configurations: PlaNet-S (A–T–), PlaNet-S (A + T–), PleNet-S (A-T +), and PlaNet-S (A + T +), which is the proposed method. Their mean IoU values (with standard deviations) are 0.71 (SD = 0.14), 0.77 (SD = 0.11), 0.73 (SD = 0.14), and 0.78 (SD = 0.10), respectively (Table 2). The IoU for PlaNet-S(A + T +) was significantly higher than that for PlaNet-S(A–T–) (*p* < 0.003) and PlaNet-S(A-T +) (*p* < 0.003), as determined by the paired t-tests with the Bonferroni-adjusted significance level; however, the difference between PlaNet-S(A + T +) and PlaNet-S(A + T–) was not statistically significant (*p* = 0.02).

**Table 2:** Intersection over Union and the absolute differences in the Counts of Connected Components for the ablation study

	IoU (mean ± SD)	CCC: matching the ground truth (%)
PlaNet-S (A-T–)	0.71 ± 0.14	7.90
PlaNet-S (A + T–)	0.77 ± 0.11	61.9
PlaNet-S (A–T +)	0.73 ± 0.14	67.9
PlaNet-S (A + T +)	0.78 ± 0.10	86.0

A and T; represents the use of data augmentation and test time augmentation, respectively. *IoU*; Intersection over Union, *CCC*; Counts of Connected Components

**Fig. 6:**
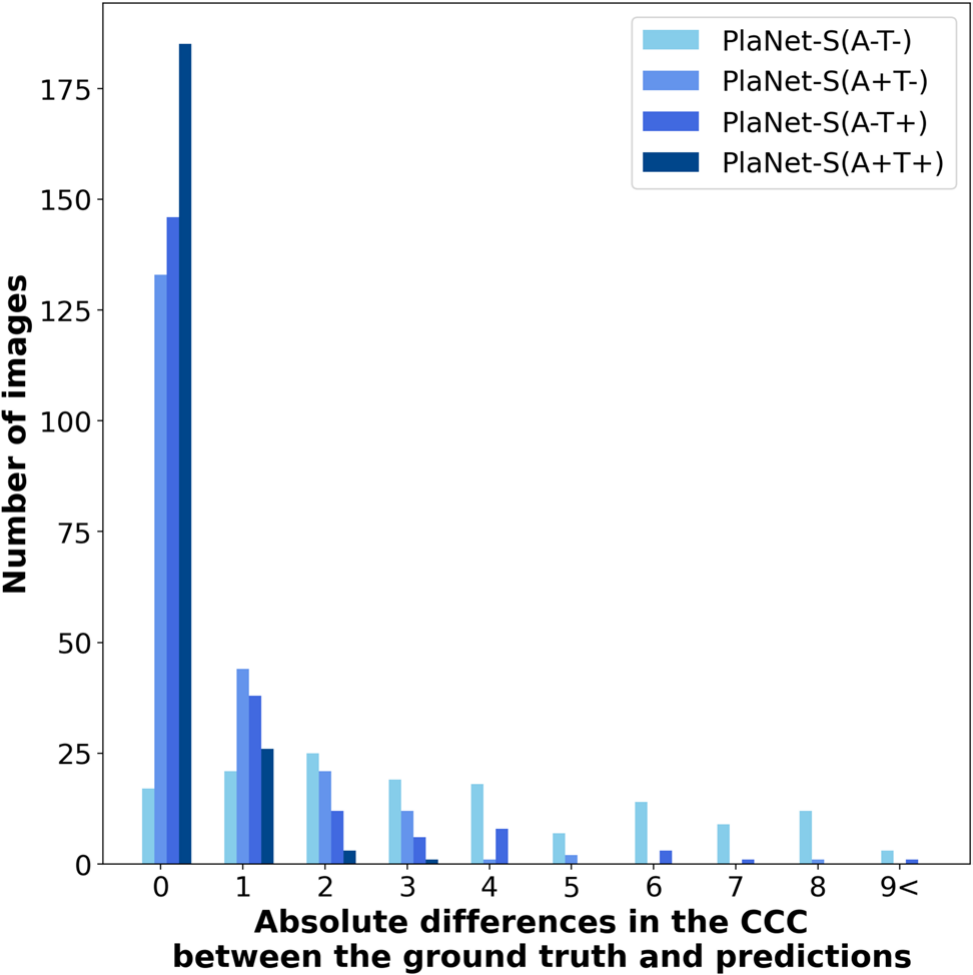
Bar plot comparing the absolute differences in the counts of connected components (CCC) between the ground truth and predictions for PlaNet-S (A–T–), PlaNet-S (A + T–), PlaNet-S (A–T +), and PlaNet-S (A + T +). The bar plot depicts the absolute differences between the ground-truth number of connected components and those inferred from the segmentation results of the four configurations. The *x*-axis represents the absolute differences in the CCC between the ground truth annotations and the segmentations performed by the DL models, whereas the *y*-axis indicates the cumulative number of test images corresponding to those differences. The CCC for PlaNet-S (A + T +) was significantly smaller than those for the other models (all *p* < 0.003), as determined by Wilcoxon signed-rank tests with the Bonferroni-adjusted significance level

Figure 6 presents a comparison of the CCC, evaluating the absolute differences between the ground-truth and inferred number of connected components for PlaNet-S (A–T–), PlaNet-S (A + T–), PlaNet-S(A-T +), and PlaNet-S (A + T +). The corresponding mean values (with standard deviations) are 17, 133, 146, and 185 test images, representing, 7.90%, 61.9%, 67.9%, and 86.0% of the dataset, respectively (Table 2). The CCC for PlaNet-S(A + T +) was significantly smaller than that for the other models (all *p* < 0.003), as determined by the Wilcoxon signed-rank tests with the Bonferroni-adjusted significance level.

### Visual Example of the Segmentation Results

**Fig. 7:**
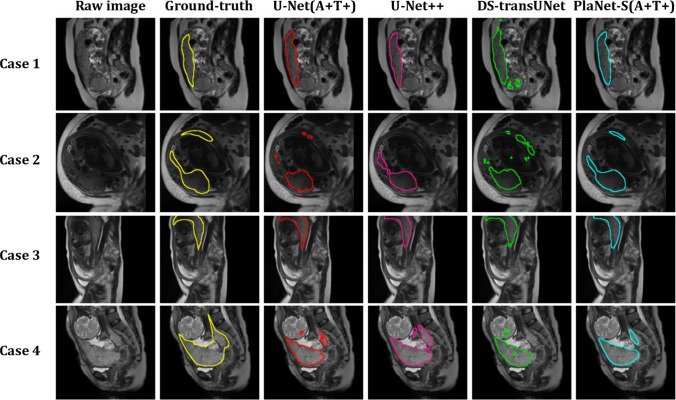
Comparative examples of segmentation results for U-Net, U-Net + +, DS-transUNet, and PlaNet-S. Figure 7 shows a comparison of the segmentation outcomes of U-Net, U-Net + +, DS-transUNet, and PlaNet-S across the four illustrative cases. In Case 1, all models demonstrated congruence with the ground truth, underscoring their potential for accurate segmentation. Case 2 highlights the superior performance of PlaNet-S, which remained consistent with the ground truth, whereas U-Net U-Net + +, and DS-transUNet exhibited a notable deviation. In contrast, Case 3 offers an instance in which PlaNet-S diverges significantly from the ground truth, whereas U-Net maintains the alignment. Finally, Case 4 provides an example in which neither model achieves concordance with the ground truth, indicating areas for further refinement

Figure 7 shows a comparison of the segmentation outcomes of PlaNet-S, U-Net, U-Net + +, and DS-transUNet across the four illustrative cases. In Case 1, four models demonstrated congruence with the ground truth, underscoring their potential for accurate segmentation. Case 2 highlights the superior performance of PlaNet-S, which remained consistent with the ground truth, whereas U-Net U-Net + + and DS-transUNet exhibited a notable deviation. By contrast, Case 3 offers an instance in which PlaNet-S diverges significantly from the ground truth, whereas U-Net maintains the alignment. Finally, Case 4 provides an example in which neither model achieves concordance with the ground truth, indicating areas for further refinement. For reference and to ensure objectivity, the segmentation outcomes for the first ten cases (Cases 5–14) from the randomized final test have been appended as supplemental material.

## Discussion

Our proposed DL model, PlaNet-S, which incorporates a heterogeneous ensemble learning approach combining both SegNeXt and U-Net, demonstrated superior segmentation results compared to conventional U-Net alone and DS-transUNet. Historically, U-Net is quite succeeded model, but there are some limitations. Researchers, investigating in segmentation task of medical images, have been tried to make U-Net better in some ways. For example, Zhou et al. proposed U-Net + +, which utilizes re-designed skip pathways and deep supervision [29]. Some researchers also make U-Net better through integrating the Transformer and attention mechanisms [30–33]. One such example is DS-transUNet [34]. However, PlaNet-S showed superiority over both U-Net + + and DS-transUNet, although the level of statistical significance varied across metrics. What makes its superiority to DS-transUNet may be transformer architecture employed in DS-transUNet, although the parameter counts between U-Net and DS-transUNet is relatively similar. Transformer-based models generally require extensive pre-training on large-scale image datasets to fully realize their performance potential [22]. In this study, because the training data are limited, the transformer-based model may not have received sufficient parameter updates for optimal learning. This likely contributed to its suboptimal performance compared to PlaNet-S. One potential reason explaining why U-Net and DS-transUNet was consistently inferior to our proposed model, PlaNet-S, is following: whereas U-Net primarily leverages local textures in images [19, 20], SegNeXt, ViT-based model, emphasizes the global shapes of objects during image recognition [21]. As the overall morphology of the placenta is typically represented as a relatively cohesive structure, appearing as one or a few consolidated masses, and its textures are marked by high-intensity signals on T2 WI, the ensemble learning approach combining SegNeXt and U-Net might be successful in capturing both these aspects. These implies the reason why U-Net + +, lacking of SegNeXt, shows inferior CCC indicators, despite the IoU for U-Net + + was comparable with that for PlaNet-S.

Our ablation study showed that data augmentation and TTA are both important for the performance of PlaNet-S. Medical images often have difficulty in data collection. Improving the generalization performance of models remains a significant challenge. Data augmentation serves as a viable technique in addressing this challenge. A similar approach applied during inference is known as TTA, which is renowned for its ability to provide robust and stable predictions by incorporating multiple augmented views of the same image. In our study, TTA was implemented during the test process to leverage its inherent strengths and enhance the accuracy of placenta segmentation, besides data augmentation during the training step. Such augmentation-driven robustness can be particularly beneficial in medical imaging, where slight variations or perspectives in imagery can affect a model’s decision. TTA contributes to improving accuracy, mitigating overfitting, and aiding in better generalization of unseen data [28]. These characteristics of TTA also play a significant role in showing a more cohesive and consistent segmentation, which might improve the CCC indicators. Our ablation study supports above mentioned idea. As mentioned in “Materials and Methods” section, in our ablation study, the abbreviations A and T denote the use of data augmentation and test time augmentation (TTA), respectively. In terms of IoU, there were no differences between PlaNet-S (A + T–) and PlaNet-S (A + T +), but PlaNet-S (A + T +) performed better than PlaNet-S (A + T–) in CCC indicators. PlaNet-S (A–T +) was inferior to PlaNet-S (A + T +), in both IoU and CCC indicators. These results showed the significance of data augmentation, and the effectiveness of TTA in CCC indicators. Consequently, TTA contributed to the improved performance of our placenta segmentation model, because the TTA has advantages with the cohesive and consistent segmentation, and the placenta is typically represented as a relatively cohesive structure, appearing as one or a few consolidated masses, as written above. However, we cannot ignore a limitation of our ablation study. We consider the ablation study depicts the role of each component, but there may be a possibility that we overlook synergies among components, which could lead to conflicting results.

The development of PlaNet-S led to significant advancements in medical imaging, potentially resolving the traditional time-consuming and specialist-dependent processes of placental segmentation [12]. The automation provided by PlaNet-S streamlines workflow and reduces the need for specialized expertise. Future research should pivot toward a fully automated diagnostic AI capable of identifying perinatal conditions such as placenta accreta, preeclampsia, and fetal growth restriction. Combined with deep-learning technologies for classifying placental diseases, PlaNet-S has the potential to form a comprehensive diagnostic model for placental disorders. As written above, some MRI-based diagnostic or predicting models have been studied [24–26] and these models require segmentation of placenta. PlaNet-S, an automatic semantic segmentation model with relatively high accuracy, can be combined with these diagnostic or predicting models. We think fully automated models, which enables segmentation and diagnosis, are needed in order to minimize the differences of diagnostic accuracy across various institutions, regardless the radiologist’s expertise. Furthermore, the principles underpinning PlaNet-S can be extended to the segmentation and diagnosis of various other organs, thereby broadening its impact on medical diagnostics.

This study has three limitations. First, the segmentation annotations were performed by a single radiologist, which may introduce potential bias due to inter-observer variability in manual segmentation. Second, the dataset was obtained from a single facility. Although our study included images acquired from four different MRI machines, sourcing data from a single facility may limit the generalizability of the model, introduce bias related to specific equipment or demographic characteristics, and increase the risk of overfitting. Future studies should prioritize diversifying the data sources and employing advanced data augmentation techniques during training to enhance placental segmentation and ensure broader applicability. Third, PlaNet-S has the limitation that it may not segment well when the placenta is thin and elongated, although this is less frequent than when using U-Net.

## Conclusions

Utilizing ensemble learning with SegNeXt and U-Net, PlaNet-S showed a higher performance on the segmentation task of the placenta than the traditional U-Net, U-Net + +, the transformer-based benchmark model, and DS-transUNet. This model addresses the challenges of time-consuming physician-assisted manual segmentation and offers the potential for diverse applications in placental imaging analyses.

## Supplementary Information

Below is the link to the electronic supplementary material.

Fig. 8 Supplementary Fig.8

High resolution image (8.50 MB)

Fig. 9 Supplementary Fig.9

High resolution image (9.42 MB)

## Data Availability

Data from this study are not publicly available and are restricted to protect patient privacy in accordance with the institutional review board guidelines. The data were securely stored under controlled access at Tohoku University Hospital. Requests for data access may be submitted to the corresponding author and considered by the IRB on a case-by-case basis, subject to an approved research proposal that meets the requisite criteria for ethical handling and privacy protection.
