# Supplementary figures and images for: PlaNet-S: an Automatic Semantic Segmentation Model for Placenta Using U-Net and SegNeXt
Source: J Imaging Inform Med. 2025 May 27;39(1):400–10. doi: 10.1007/s10278-025-01549-9 (PMC12920942; doi:10.1007/s10278-025-01549-9)

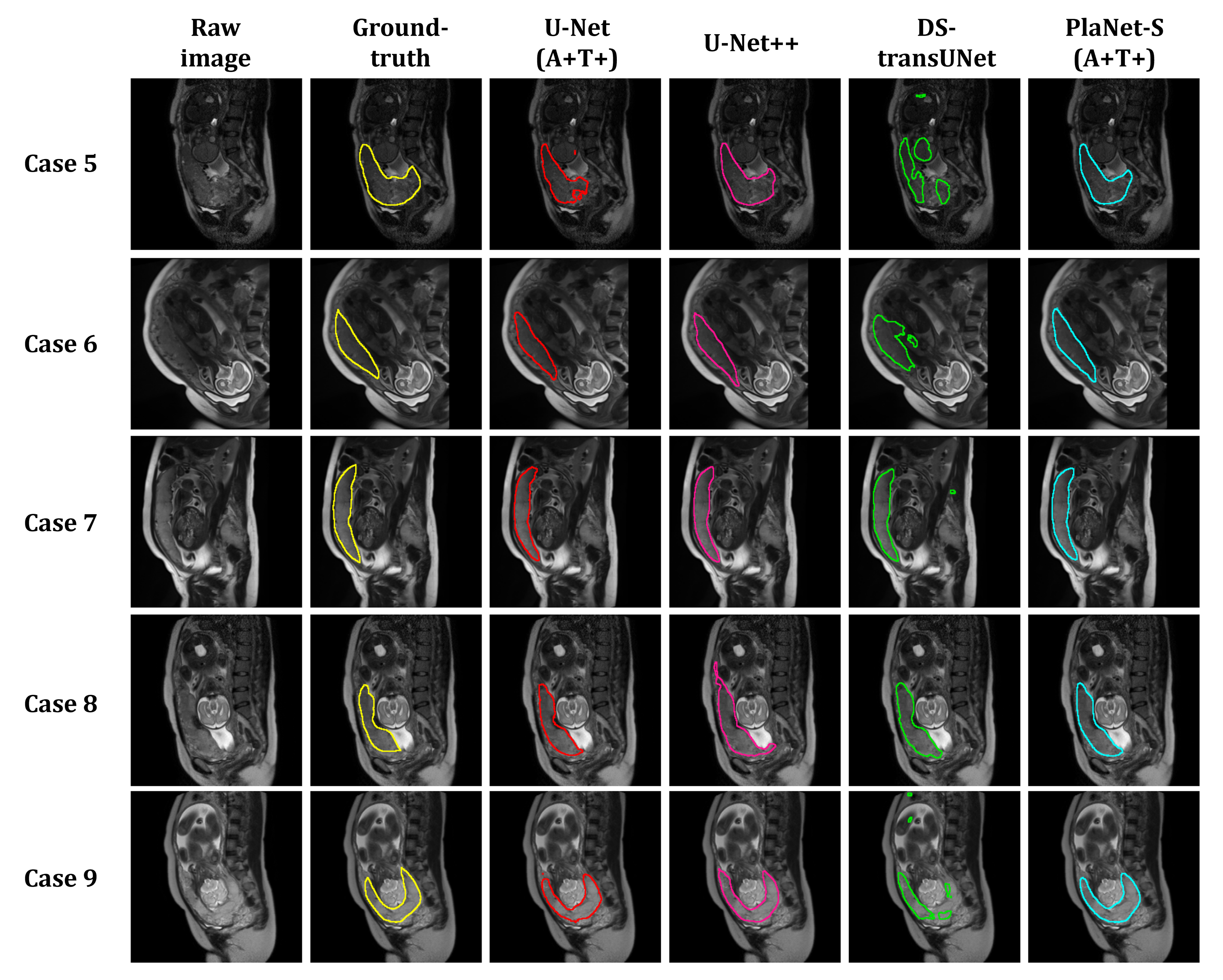

Supplement: Supplementary file 1 — Supplementary Fig.8 [file 10278_2025_1549_Fig8_ESM.png]

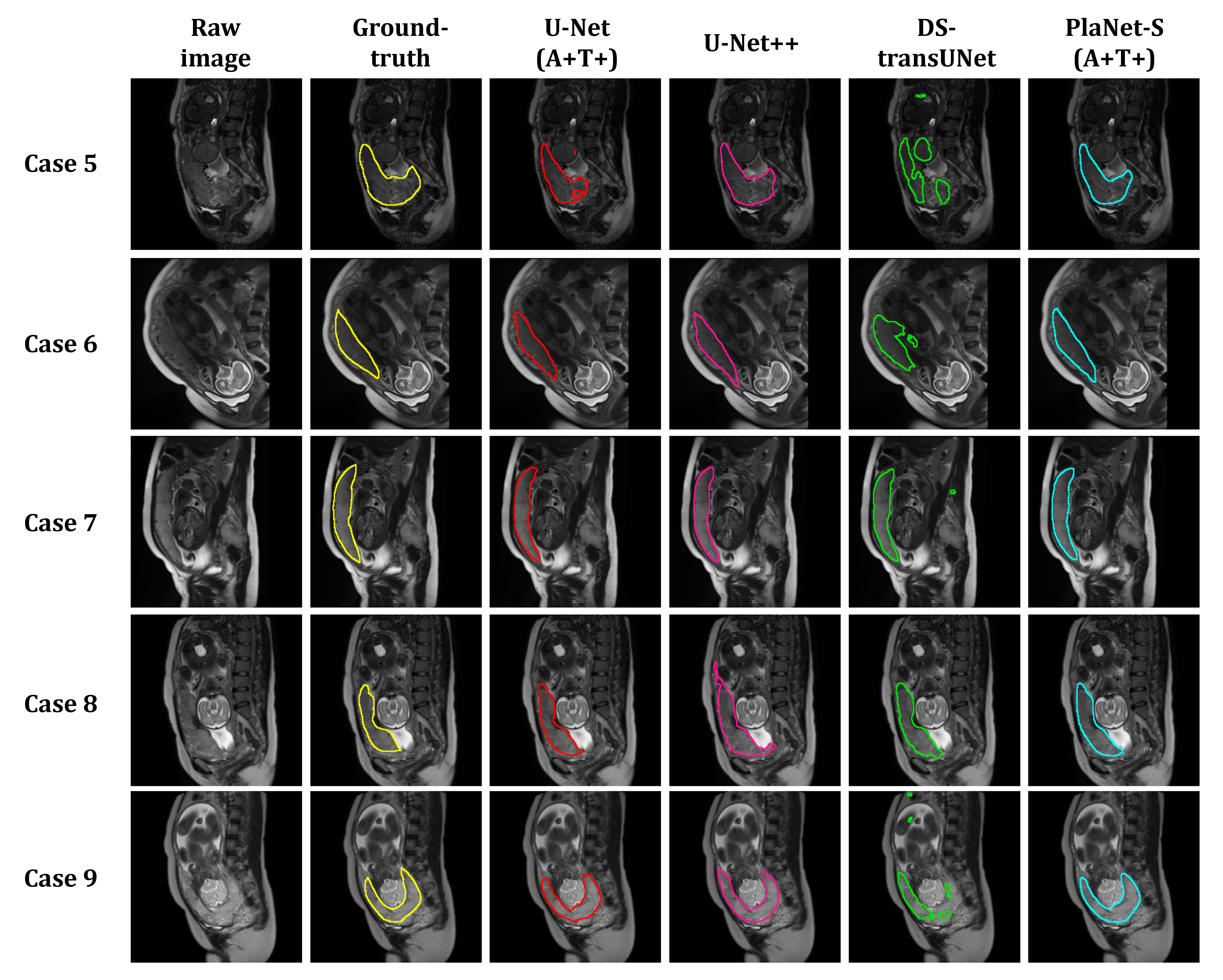

Supplement: Supplementary file 2 — High resolution image (8.50 MB) [file 10278_2025_1549_MOESM1_ESM.tif]

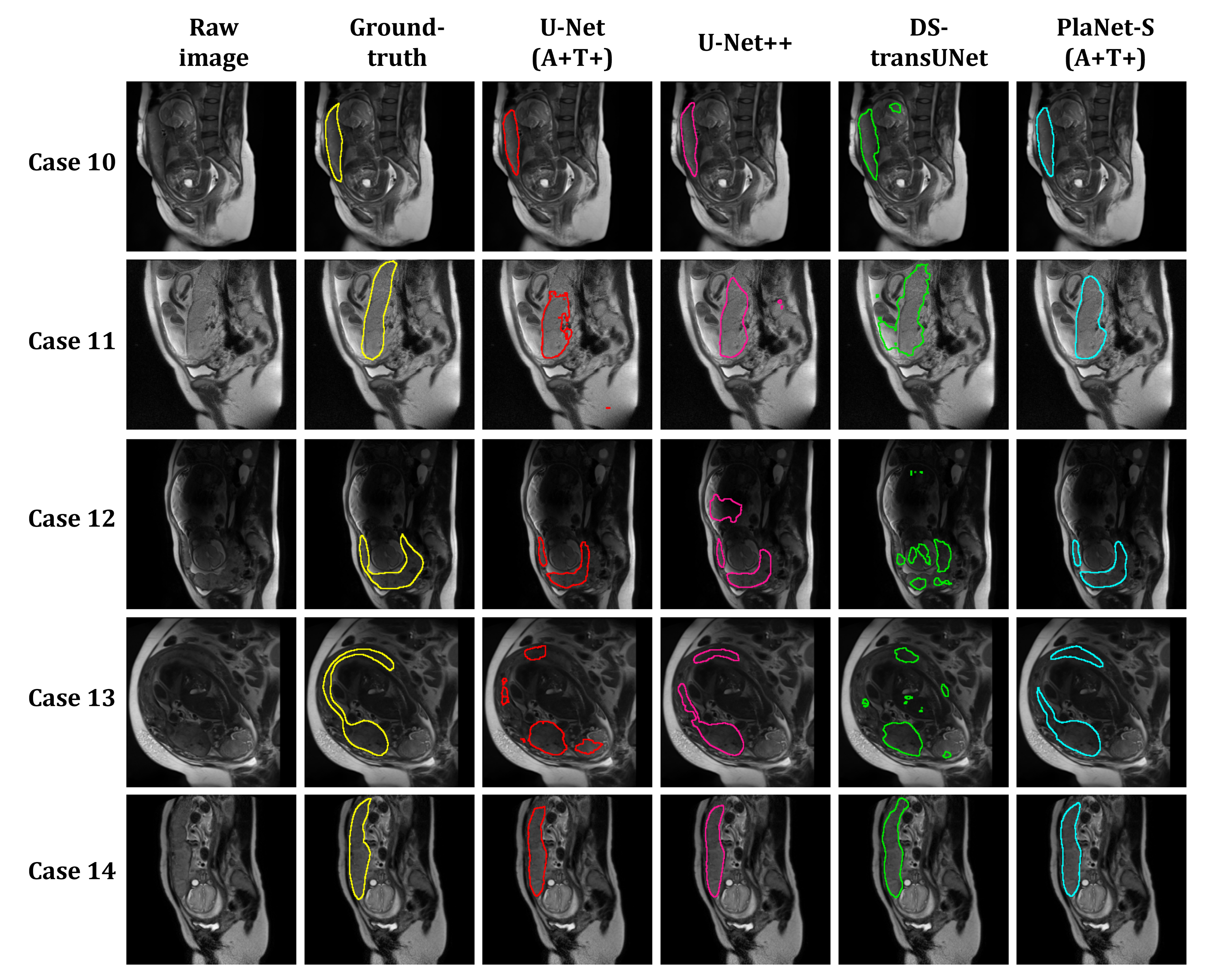

Supplement: Supplementary file 3 — Supplementary Fig.9 [file 10278_2025_1549_Fig9_ESM.png]

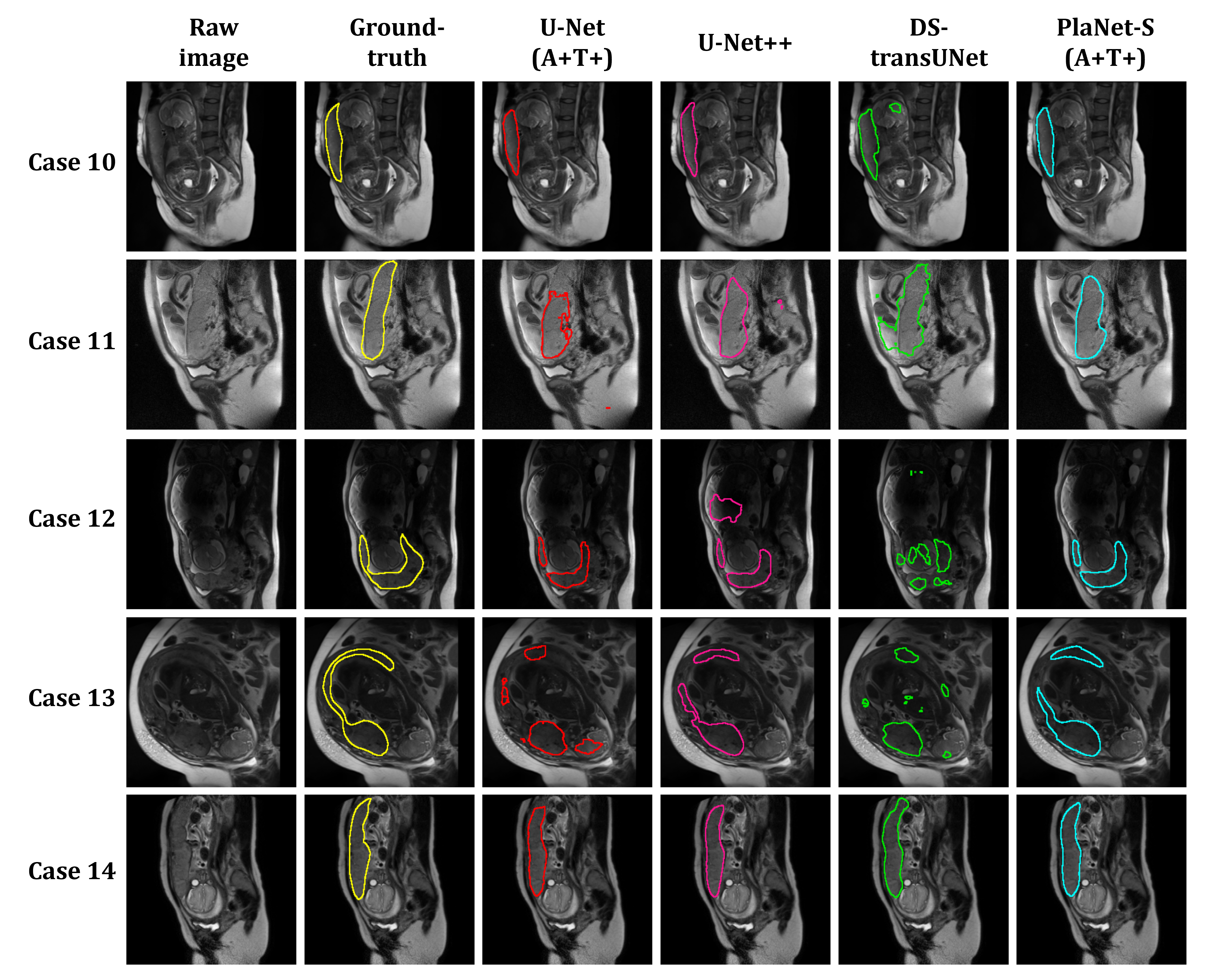

Supplement: Supplementary file 4 — High resolution image (9.42 MB) [file 10278_2025_1549_MOESM2_ESM.tif]
